# Sensory regimes of gentrification: urban transformation, embodied experience, and neoliberal spatial production in Cihangir

**DOI:** 10.3389/fsoc.2026.1871741

**Published:** 2026-10-19

**Authors:** Hasan Basri Kartal, Asiye Nisa Kartal

**Affiliations:** 1 Department of Architecture, Mimar Sinan Fine Arts University, Istanbul, Türkiye; 2 Department of Architecture, Istanbul Technical University, Istanbul, Türkiye

**Keywords:** *sensewalking*, sensory displacement, sensory urban transformation, *soundscape*, urban *smellscape*

## Abstract

Gentrification has predominantly been examined through economic restructuring, demographic change, and spatial reorganization, while its sensory and embodied dimensions remain underexplored in urban sociology. This study reconceptualizes gentrification as a multi-layered urban transformation regime operating not only through material and economic change but also through the reconfiguration of sensory urban experience. Addressing the visual bias in urban research, it develops a multi-phase sensory-ethnographic framework to examine how market-driven transformation shapes everyday sensory environments and lived experiences of place. The empirical design combined structured group and solo *sensewalking* with local residents in Cihangir and a supplementary couple *sensewalking* with Istanbul-based architects academically familiar with the neighborhood's transformation. The later field encounter followed a different route and expanded documentation through photography, video, sound recording, and an in-walk walking interview. The field encounters were analyzed as related but non-equivalent forms of situated knowledge. Resident-based findings identified curated cafe clusters, differentiated *soundscape*s and *smellscape*s, nodal sensory intensity, and selective forms of belonging. The photographic and walking-interview material documented the persistence of commercial curation while also revealing steep topographic transitions, unexpected views, residual artistic memory, social heterogeneity, preserved shopfronts, historical traces, intensified media visibility, and the coexistence of everyday sociability with waste, traffic, crowding, and movement friction. These materials refine sensory homogenization as a partial and uneven process produced through selective intensification rather than complete neighborhood-wide standardization. The study argues that gentrification should be understood not only as spatial displacement but also as sensory displacement, through which familiar environments are perceptually reorganized while residual material and sensory traces continue to structure attachment. By integrating embodied, temporal, multimodal, and participant-narrated field encounters, the study shows how spatial production, classed belonging, and urban inequality are reproduced through everyday sensory environments under neoliberal conditions.

## 1 Introduction

Gentrification has largely been examined through economic restructuring, demographic change, housing-market dynamics, and spatial reorganization (Shaw, 2008; Osman, 2016; Yeom and Mikelbank, 2019; Redfern, 2003; Slater, 2011). These perspectives have significantly advanced understandings of urban transformation by revealing how capital investment, class restructuring, and policy interventions reshape cities (Scott, 2001; Krätke, 2014). Yet, despite this substantial body of knowledge, gentrification remains largely understood through material, economic, and visual indicators, while its sensory dimensions remain comparatively underexplored (Degen, 2008; Degen and Rose, 2012; Tiainen et al., 2019; Järviluoma and Murray, 2023). Existing accounts frequently focus on who is displaced, how property values change, and how neighborhoods are physically transformed, but pay less attention to how urban environments are experienced, perceived, and inhabited in everyday life (Kenny, 2014; Low, 2015; Wankhede et al., 2023).

This limitation is particularly significant because urban transformation does not merely alter the physical and social composition of neighborhoods; it also reshapes the sensory conditions through which urban life is experienced (Degen, 2017, 2018; Järviluoma and Murray, 2023). Changes in *soundscapes* (the combination of sounds in the environment) (Schafer, 1993), *smellscape*s (the olfactory environment of urban places) (Henshaw, 2013; Davis and Thys-Senocak, 2017), atmospheres, urban rhythms, and patterns of social interaction frequently accompany processes of urban redevelopment and neighborhood change (Degen, 2008; Low, 2015; Tiainen et al., 2019; Jackson et al., 2021). Such transformations influence how residents experience belonging, familiarity, attachment, and exclusion within changing urban environments Borer, (2013; Low, 2015; Howes, 2019; Abram, 2023). These transformations are sensory-spatial as well as socio-political and physical, shaping everyday experiences of place and influencing how urban change is perceived and experienced (Degen and Rose, 2012; Tiainen et al., 2019; Järviluoma and Murray, 2023; Di Croce, 2025).

Addressing these questions requires moving beyond the visual bias that has historically characterized much urban research. Urban life unfolds through multisensory engagements involving sight, sound, smell, touch, and movement, all of which contribute to meaning, memory, and place attachment (Henshaw, 2013; McLean, 2019; Low, 2015; Tiainen et al., 2019). Nevertheless, urban studies have long privileged visual representations and observable spatial forms, often neglecting the embodied and non-visual dimensions of urban experience (Degen, 2008, 2018; Degen and Rose, 2012; Mattern, 2008; Järviluoma and Murray, 2023). Recent scholarship has increasingly challenged this *ocularcentric* orientation (Bull et al., 2006) by emphasizing embodied methodologies and multisensory approaches to understanding urban environments (Lucas and Romice, 2008; Henshaw and Cox, 2009; Pink, 2009; Low, 2015; Parker et al., 2024, 2026). These approaches suggest that urban places are not only seen but also heard, smelled, touched, and rhythmically experienced, making sensory perception a critical dimension of urban transformation.

While sensory urbanism has expanded considerably over the last decade (Zardini, 2012), a significant gap remains in understanding how market-driven gentrification produces sensory environments and embodied urban experiences at the neighborhood scale, and how these environments persist, shift, or are reinterpreted across different field moments. Existing studies have shown the importance of sensory perception in shaping urban experiences, yet relatively little attention has been devoted to how sensory environments are reorganized within fragmented, consumption-oriented processes of urban transformation. The relationship between gentrification and sensory experience remains insufficiently theorized and empirically investigated, particularly when sensory change is treated as spatially uneven and temporally situated rather than as a uniform neighborhood condition. Understanding urban transformation, therefore, requires attention not only to changing social and spatial structures but also to the sensory conditions through which people encounter, remember, and live these changes.

This study addresses this gap by developing a sensory-ethnographic framework for analyzing gentrification as a multi-layered urban transformation regime operating simultaneously at material, symbolic, and sensory levels. The study examines Cihangir (Istanbul), a neighborhood that has undergone substantial gentrification through a fragmented, predominantly market-driven trajectory accelerated by cultural representation, symbolic capital, and consumption-oriented urban dynamics (Yetiskul and Demirel, 2018; Uzun, 2020). Rather than treating Cihangir merely as an empirical case, the study approaches it as a temporally layered sensory regime in which market forces, localized governance, cultural production, historical continuity, and everyday practices become perceptible in urban experience. The later 2026 field encounter was incorporated not as a statistically equivalent second sample, but as a temporal and expert-informed extension of the 2025 resident-based fieldwork.

The study adopts a sensory-ethnographic and mobile research design centered on *sensewalking* (McLean, 2019; Parker et al., 2024). *Sensewalking* enables the exploration of multisensory urban experiences by engaging participants in structured walking sessions through changing urban environments (Henshaw and Cox, 2009; Vasilikou, 2019; Shookouhi Dolat Abadi and Zarei, 2021). Through embodied movement, participants actively engage with sounds, smells, textures, atmospheres, movement conditions, and the tempo of urban life, generating insights into how urban transformation is experienced perceptually. In the present study, the 2025 group and solo walks were complemented by a 2026 paired walk that combined the same sensory attention with photographic, video, and sound recording and a walking interview. This approach aligns with broader methodological shifts toward mobile and experiential research that emphasize walking as a means of accessing embodied knowledge, spatial perception, and *in-situ* interpretation (Degen and Rose, 2012; Rubidge and Stones, 2009).

The study addressed two interrelated research questions. First, how does market-driven gentrification transform Cihangir beyond visual dimensions? Second, the complementary resident-based and expert-informed encounters revealed convergent and contrasting sensory patterns that refined the interpretation of Cihangir as a selectively intensified, materially layered, and socially differentiated sensory regime. In responding to these questions, the study moves beyond visually descriptive and structurally deterministic accounts of gentrification. It reconceptualises urban transformation as a multi-layered and temporally situated process operating simultaneously through material restructuring, symbolic production, sensory reconfiguration, and the selective intensification of particular urban interfaces. By linking sensory reconfiguration to classed belonging, everyday public-space use, and the social production of space, the study speaks directly to urban-sociological debates on gentrification and inequality.

## 2 Conceptual framework

### 2.1 Sensory urban experience beyond visual perception

*Ocularcentrism* refers to the epistemological, cultural, and methodological privileging of vision over other sensory modalities in producing knowledge and experience. By this visual hegemony, other sensory modes—such as hearing, smell, touch, and taste—have often been relegated to secondary or less reliable forms of perception. In Lynch (1960) and Cullen (1961), this visual bias was reinforced by framing urban legibility, imageability, and townscape experience primarily through visual coherence and spatial aesthetics (Lanci, 2022; Brackenbury, 2023; Jones, 2025). This visual hegemony has not only structured how cities are studied but also how they are governed, planned, and commodified, privileging what is visible and representable while marginalizing embodied sensory dimensions of urban experience.

A sensory approach to urban experience provides a nuanced understanding of how cities are lived and felt. They are not universally shared; they are shaped by individual differences, cultural backgrounds, and social positions (Low, 2015; Howes, 2019, 2022; Degen, 2017; Cheng and Bowring, 2024). By foregrounding the body and its sensory capacities, this approach challenges visual-centric paradigms and highlights the importance of multisensory engagement in shaping urban places. The concept has emerged as a critical lens for understanding how cities are perceived, navigated, and lived (Lucas and Romice, 2010; Low, 2015; Abusaada, 2020; Peca Amaral Gomes, 2023). Urban experiences are deeply relational, involving interactions between individuals, material environments, and social contexts. Moving beyond purely visual interpretations of urban space, scholars increasingly emphasize the multisensory dimensions of urban space—how sound, smell, touch, and even taste contribute to meaning and memory in cities. This shift reflects a broader epistemological move within urban studies toward embodied and experiential approaches to place (Howes and Classen, 1991; Howes, 2003, 2019, 2022).

(Borer 2013) emphasizes that being in the city is not merely a spatial condition but an experiential one, shaped by sensory engagements. The sensory city underscores the importance of designing cities that engage multiple senses (Adams et al., 2009). Sensory considerations in urban environments suggest that conventional research tools—maps, diagrams, and visual renderings—cannot capture the complexity of sensory environments. Innovative representational techniques, including narrative descriptions, participatory mapping, and immersive simulations, may convey the lived experience of urban spaces. Low (2015) argues that such approaches overlook the richness of everyday sensory encounters that shape how individuals engage with urban environments. Mattern (2008) highlighted how urban design mediates non-visual senses. She argued that cities are frequently designed with a bias toward visual aesthetics, rendering other sensory dimensions invisible in urban discourse. This neglect can lead to environments that are visually appealing but sensorially overwhelming or alienating. Sensory methodologies offer valuable tools for uncovering diverse urban experiences (Järviluoma and Murray, 2023; Parker et al., 2024, 2026). They foreground the body as a key instrument of knowledge production, allowing researchers to capture fleeting, often overlooked dimensions of urban experience.

Early urban research tended to privilege sight as the dominant mode of perception, aligning with modernist planning traditions that emphasized visual order, legibility, and form. Subsequent studies argued that *ocularcentrism* produces an incomplete and selective understanding of urbanity by reducing lived environments to visual surfaces and representational forms. This perspective suggests that urban experience emerges through multisensory entanglements rather than visual observation alone.

### 2.2 Sensory transformation under gentrification

In urban transformation processes such as gentrification, sensory experiences increasingly become instruments of neoliberal governance. Thus, moving beyond *ocularcentrism* is not simply a methodological expansion but a theoretical necessity for understanding how gentrification operates through the sensory organization of spatial production, politics, and everyday urban practices (Degen, 2008, 2018; Degen and Rose, 2012; Tiainen et al., 2019). Within neoliberal urban policies, the neoliberal city is characterized by the increasing commodification of urban space, reconfigured as an object of investment, competition, and speculation. Public spaces are restructured as sites of consumption, and urban environments are strategically redesigned to attract capital and desired users. However, this process is not limited to economic restructuring; it also entails producing market-oriented sensory environments whose intensity and visibility may be concentrated on particular streets, nodes, and interfaces rather than distributed evenly across a neighborhood. At the same time, the state's role becomes more complex. Rather than withdrawing from urban processes, the state is reconfigured as a regulatory, facilitative, and enabling actor that shapes the institutional and legal conditions under which gentrification unfolds (Brenner and Theodore, 2005; Herzfeld, 2010; Pinson and Morel Journel, 2016). Urban transformation emerges through hybrid and context-specific configurations of state, market, civil society, and everyday actors. From a sensory perspective, these arrangements raise questions not only about what changes, but also where, when, and through which perceptual conditions change becomes noticeable.

This study proposed a conceptual intervention. Gentrification was approached not merely as a process, but as a multi-dimensional urban transformation regime that operates simultaneously at material, symbolic, and sensory levels. Gentrification extends beyond the transformation of physical space and becomes directly linked to the reproduction and reorganization of sensory experiences. Urban space functions as both a site of economic value production and a representational and sensory arena where symbolic and cultural values circulate. These processes shape the sensory coding of space, influencing atmosphere, movement, interaction, and the boundaries of appropriate use. Through these processes, urban places are not only reformed but also re-sensed (Degen, 2008; Jackson et al., 2021; Järviluoma and Murray, 2023). The concept of a sensory regime therefore describes not a stable sensory totality, but a patterned and uneven organization of sensory cues, practices, and atmospheres that can persist, intensify, or be reinterpreted over time.

Shifts in auditory and olfactory experiences, everyday spatial practices, movement conditions, and atmospheric qualities accompany urban transformation. These changes produce new forms of urban experience, while older material and sensory traces may remain perceptible within the same environment. Thus, gentrification operates as a dual process of transformation at both the material and sensory levels (Abram, 2023; Di Croce, 2025), but sensory transformation should not be assumed to erase continuity. Research on temporal and experiential urban change demonstrates that familiarity, memory, and sensory attachment can persist through residual cues even when the wider meaning of a place is being reorganized (Degen, 2017, 2018; Järviluoma and Murray, 2023). This study's conceptual structure therefore shifted the focus to how urban environments are constructed, experienced, and transformed through specific configurations of power and governance, and how selective sensory intensification coexists with historical and everyday continuity. Cihangir was treated not simply as an empirical case, but as a site through which a market-driven urban transformation regime and its temporally layered sensory configuration were examined.

The concept of gentrification was first introduced in the 1960s to describe class-restructuring processes observed in urban centers. The classical definition focused on the spatial and demographic transformations that occur when middle-class groups move into urban places previously inhabited by lower-income populations (Shaw, 2008; Osman, 2016; Yeom and Mikelbank, 2019). However, this early formulation framed gentrification within a relatively limited context, treating it primarily as a localized and materially observable process.

As capitalist production relations transformed, cities shifted within the global system, and spatial processes became more financialized, the concept of gentrification expanded significantly. It began to be situated within a much broader analytical framework that conceptualizes urban transformation as a multi-scalar, globally embedded phenomenon (Scott, 2001; Krätke, 2014). The literature began to understand gentrification not merely as a housing-market-driven process, but as a complex restructuring mechanism emerging at the intersection of capital accumulation, spatial revaluation, financialization, cultural production, and urban rebranding. Yet, gentrification has largely been analyzed through economic, spatial, and policy-oriented lenses, often neglecting its experiential and perceptual dimensions (Redfern, 2003; Slater, 2011).

This study argues that such approaches remain insufficient because they overlook the transformation of sensory urban experiences. From this perspective, gentrification should be understood not only as a restructuring of place but also as a process that reorganizes embodied experience. Urban transformation does not simply alter who lives in a neighborhood or how property values change; it also reconfigures how space is heard, smelled, touched, navigated, remembered, and emotionally perceived (Kenny, 2014; Wankhede et al., 2023). This study conceptualized gentrification as a sensory urban transformation regime: a multi-layered and spatially uneven process through which perceptual environments, everyday practices, and atmospheres are reorganized. In this sense, urban places become not only socio-political constructs but also sensory fields in which meanings, identities, power relations, and degrees of familiarity are materially and experientially produced.

## 3 Methodology

This study adopted a qualitative, single-case, multi-phase, and sensory-ethnographic research design operating across spatial, social, sensory, and temporal dimensions. The study integrated a sensory urban experience perspective with an empirical mobile methodology, enabling the investigation not only of how gentrification was produced and represented, but also of how it was experienced and re-encountered at the sensory level (see Figure 1). The first field phase comprised the 2025 group and solo *sensewalking* with local residents, while the second comprised a July 2026 couple *sensewalking* with two architects. The later walk was designed as a complementary temporal and expert-informed extension rather than an equivalent replication. Figure 1 summarizes the progression from conceptualization and case selection to the two field phases, multimodal data collection, within-phase thematic analysis, cross-phase interpretation, and the development of the study's sensory urban transformation framework.

**Figure 1:**
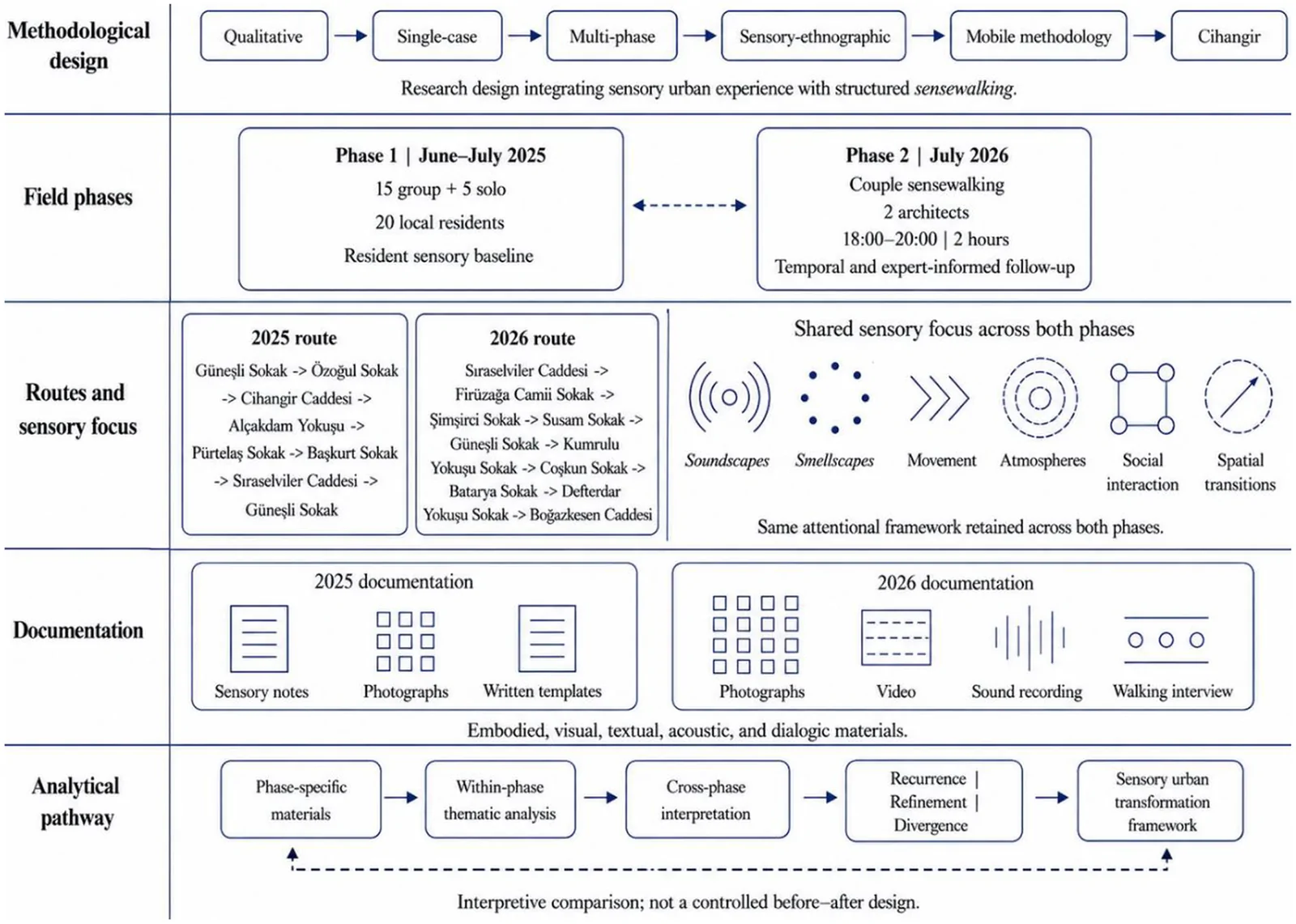
Overall multi-phase research design. Source: Own elaboration.

The second field phase was undertaken to revisit and refine the sensory patterns identified through the 2025 resident-based walks. The first phase revealed that sensory curation, continuity, displacement, and social belonging were unevenly distributed across Cihangir and concentrated at particular urban nodes. Returning to the neighborhood in July 2026 through a second route and a paired, expert-informed walking configuration enabled the study to examine whether these patterns remained perceptible in a later field encounter and how they could be reconsidered through dialogic and multimodal documentation. The 2026 phase was therefore designed as an analytical and temporal extension of the 2025 fieldwork, rather than as a replication or a controlled before-and-after comparison.

### 3.1 Cihangir as a temporally layered case

The study adopted an in-depth, temporally extended single-case design centered on Cihangir, a neighborhood that has undergone substantial gentrification under fragmented, predominantly market-driven conditions. The case was selected to examine how a specific urban transformation regime produces sensory environments and embodied urban experiences, and how these sensory configurations remain continuous or become differently perceptible across later field encounters. The single-case design enabled close attention to the neighborhood's historical layers, spatial transitions, everyday practices, and sensory variations rather than treating transformation as uniform across the district. The 2026 follow-up strengthened this place-specific approach by revisiting Cihangir through a second route and a different participant configuration.

Cihangir represents a predominantly market-driven and fragmented trajectory of urban transformation. This trajectory unfolded within the wider neoliberal restructuring of Istanbul's property markets and urban governance (Lovering and Türkmen, 2011; Polat, 2021). Previous research has shown that the neighborhood has experienced significant socio-spatial restructuring through processes of cultural rebranding, symbolic value production, consumption-oriented redevelopment, and middle-class reinvestment (Uzun, 2003; Potuoglu-Cook, 2006; Hoffmann, 2007; Wildner and Kokot, 2008; Salgamcioglu and Ünlü, 2014; Demirel, 2015; Yetiskul and Demirel, 2018; Uzun, 2020; Özbay Kinaci, 2014; Uluengin, 2023). These processes have transformed Cihangir into one of Istanbul's most prominent examples of neighborhood change associated with lifestyle consumption, cultural representation, and urban revalorization. At the same time, its dense residential fabric, steep and narrow streets, concentration of cafes and small-scale commercial venues, historic and religious traces, greenery, active public life, and walkable but spatially compressed street network provide a suitable environment for investigating both the curation and the persistence of sensory urban conditions. The two field phases used these layered conditions as the empirical ground for examining how sensory transformation is concentrated, negotiated, and encountered in everyday movement.

### 3.2 *Sensewalking* as a sensory-ethnographic method

Walking plays a central role in sensory urban research. Walking enables an embodied engagement with the city, activating perceptual memory and facilitating a dynamic interaction between past experiences and present stimuli (Zacharias, 2001; Matos Wunderlich, 2008; Degen and Rose, 2012; Vasilikou, 2019; Murray and Järviluoma, 2020). To capture the experiential and perceptual dimensions of urban transformation, the study employed *sensewalking* as its primary data collection method. *Sensewalking* is a qualitative, mobile, and embodied research approach grounded in phenomenological and urban ethnographic traditions (Henshaw and Cox, 2009; Degen and Rose, 2012; Henckel, 2019; Ghaderi and Khatami, 2023; Parker et al., 2025; Torriani et al., 2026). It enabled the exploration of multisensory urban experiences by engaging participants in structured walking through specific urban environments. Unlike conventional methods that prioritize visual analysis, *sensewalking* foregrounds the olfactory, auditory, tactile, atmospheric, and kinesthetic dimensions of urban space, while walking itself serves as both a mode of data production and a form of experiential immersion.

*Sensewalking*-based research has been developed across multiple disciplines to investigate how sensory perception, multisensory integration, and embodied movement shape human experience of place. Foundational work conceptualized sensory experience as the result of both individual sensory stimuli and their integration into *sensescape*s, emphasizing that combined sensory inputs produce qualitatively different experiences than isolated stimuli. Research has also combined walking with *in-situ* conversational techniques, including *sensetalk*s and semi-structured interviews, to document how smell, sound, touch, movement, and social interaction collectively construct a *sensescape* (Gomes, 2017; Henckel, 2019; Ba et al., 2023). These approaches are particularly relevant for transforming neighborhoods because they enable perception and interpretation to be articulated as changing spatial and sensory conditions are encountered.

The 2025 field phase used group and solo *sensewalking* to distinguish shared observations from more individualized sensory encounters. The July 2026 follow-up introduced what this study terms *couple sensewalking*: a paired walk in which two participants moved through the neighborhood together, exchanged observations in motion, and took part in a walking interview. This configuration was not treated as a new standardized method or as a direct substitute for the earlier resident sample. Instead, it provided a complementary dialogic layer in which two walkers could negotiate sensory and spatial observations as they encountered the same urban sequence. Because both participants were architects living in Istanbul and academically knowledgeable about Cihangir's transformation, the later walk also added an expert-informed reading of street sections, building interfaces, topography, historical traces, and the relationship between material form and atmosphere.

Multisensory walking practices have also employed creative and multimodal forms of documentation, including photography, sensory notation, sound recording, tactile observation, and reflective mapping (Sakantamis, 2015; Parker et al., 2024; Jiang et al., 2024; Parker et al., 2025; Torriani et al., 2026). Previous research has examined *soundscape*s (Henckel, 2019, 2021; Parker and Spennemann, 2022; Huang and Kang, 2015; Tiainen et al., 2019), olfactory environments (Spennemann et al., 2023; Torriani et al., 2026), gustatory experience (Parker et al., 2026), and tactile perception (Parker et al., 2024), although these modalities are often studied separately; their interaction remains central to the experience of urban environments (Zhong et al., 2022). The present study retained an integrated sensory orientation across both field phases. The 2026 addition expanded the documentation through 106 photographs, video recording, sound recording, and a walking interview, allowing the later field encounter to register material, spatial, temporal, and conversational dimensions alongside the established sensory framework.

### 3.3 Multi-phase fieldwork and data collection

The empirical component of the study was organized in two related field phases designed to capture multisensory urban experiences in Cihangir. Phase 1 was undertaken in June and July 2025 and comprised structured group and solo *sensewalking* with 20 local residents. Phase 2 was conducted in July 2026 as a supplementary *couple sensewalking* with two architects living in Istanbul and academically familiar with Cihangir's transformation. The phases shared the same substantive attention to *soundscape*s, *smellscape*s, movement, atmosphere, social interaction, spatial transitions, and the relationship between sensory perception and urban change, but they differed in route, participant profile, and documentation format. The second phase therefore extended the fieldwork temporally but was not treated as an equivalent longitudinal sample.

In the 2025 phase, 15 participants took part in group *sensewalking* and five participants conducted solo *sensewalking*. All 20 participants were local residents aged between 25 and 69, with varying levels of familiarity and everyday engagement with the neighborhood (Pearce, 2012). Group walks enabled collective recognition and discussion of recurring sensory patterns, while solo walks enabled more individualized attention to subtle transitions, moments of discomfort, and perceptual discontinuities. We recruited participants through an open call announced via social media.

Before the 2025 and 2026 sessions, participants received information about the study procedures and provided informed consent. They were also asked whether any disability or sensory- or mobility-related condition might affect their participation; none reported a condition affecting mobility, hearing, vision, smell, or tactile perception during the walks.

From a sensory-ethnographic perspective, the 2025 route was selected to maximize exposure to diverse multisensory conditions, including variations in *soundscape*s, *smellscape*s, movement, density, land use, and atmospheric qualities. Including both high-density and relatively quieter areas enabled participants to experience contrasts within the neighborhood. Defining a fixed yet flexible route supported comparison across participants while preserving individual variation in perception and interpretation. The route functioned not merely as a path of movement, but as a structured experiential framework through which participants could observe, record, and interpret the sensory dimensions of urban transformation. It was divided into multiple stops representing differences in spatial enclosure and openness, residential and commercial use, intensity of social interaction, and perceptual character.

The 2025 Cihangir route followed a loop through dense residential streets, cafe clusters, and small-scale commercial nodes. It began and ended at Cihangir Park and included Sadik Pasha Mansion, Rainbow Steps, Alçakdam Yokuşu, Pürtelaş Street, Başkurt Street, and Sirkeci Mustafa Aga Mosque. The loop exposed participants to quieter residential segments, steep embodied transitions, visually distinctive landmarks, active social environments, and consumption-oriented street interfaces. This route produced the baseline field account used to identify the neighborhood's nodal sensory intensities and contrasts.

The July 2026 couple *sensewalking* followed a second Cihangir route through Siraselviler Street, Firüzaga Camii Street, Simşirci Street, Susam Street, Güneşli Street, Kumrulu Yokuşu Street, Coşkun Street, Batarya Street, Defterdar Yokuşu Street, and Bogazkesen Street. The walk lasted approximately 2 h, from 18:00 to 20:00, and was undertaken by two architects living in Istanbul who possessed academic knowledge of Cihangir's transformation. The same themes that structured the earlier *sensewalking* were retained, but the paired format enabled continuous dialogue and a walking interview during movement. We recorded photographs, video, and sound throughout the walk. The photographic dataset documents active commercial frontages, outdoor seating, planter-defined edges, narrow pavements, parked vehicles, taxis, scooters, bollards, waste containers, graffiti, older residential facades, historic masonry, religious structures, mature vegetation, and quieter side streets. The walking interview added *in-situ* reflections on topography and views, everyday coexistence, artistic and cultural memory, old shopfronts, neighborhood desirability, media visibility, and sensory pressures associated with crowding, waste, traffic, noise, and compressed street space.

The temporal organization of the fieldwork also differed across the two phases. The 2025 walks were conducted in the morning, afternoon, and evening in June and July, allowing daypart variation to enter participant accounts. The 2026 walk was a single early-evening encounter between 18:00 and 20:00 in July. This later timing made the overlap of commercial activity, pedestrian movement, outdoor social use, and vehicular circulation particularly visible in the photographic record. The 2026 field phase therefore broadened the study's temporal reach by 1 year, but it did not constitute a controlled before-and-after design because the route and participant profile differed.

Across both phases, data collection focused on how sensory experience emerged through the interaction of material setting, movement, social activity, and changing urban rhythms. During the 2025 walks, participants recorded multisensory observations, took photographs, and completed written templates containing open-ended questions and reflections. During the 2026 couple walk, the same attentional framework was maintained, but the documentation expanded to include 106 photographs, video and sound recordings, and a walking interview (see Tables 1 and 2). The photographic record was especially important for documenting material interfaces and spatial conditions that framed sensory experience. Still images were not treated as direct evidence of sound or smell; rather, they were read as visual-material contexts that could be compared with the earlier participant accounts of acoustic, olfactory, atmospheric, and embodied experience.

**Table 1:** Data collection and multi-phase fieldwork procedure.

Phases	What?	How?	Why?
Methodology design	Multi-phase sensory-ethnographic fieldwork using structured *sensewalking*	Single-case Cihangir study combining 2025 resident walks and a 2026 couple follow-up	Capture sensory transformation together with temporal continuity and difference
Fieldwork phase 1	June–July 2025 group and solo *sensewalking*	20 local residents: 15 group + 5 solo; ages 25–69	Establish a resident-based sensory baseline
Fieldwork phase 2	July 2026 couple *sensewalking*	2 Istanbul-based architects; academically familiar with Cihangir; 18:00–20:00; 2 h	Add a later, expert-informed and dialogic field layer
Routes	Two Cihangir walking routes	2025 loop route + 2026 second route	Compare recurring patterns across different urban sequences without assuming route equivalence.
Sensory focus	*Soundscape*s–*mellscape*s–Movement–Atmospheres–Social interaction–Spatial transitions	Same attentional framework retained across both phases	Maintain conceptual continuity between field encounters
2025 documentation	Sensory notes–Photographs–Written templates	Open-ended reflections + structured prompts	Integrate embodied, visual, and textual data
2026 documentation	Photographs–video–sound recording–walking interview	Continuous paired walk and multimodal recording	Document material, temporal, acoustic, and dialogic dimensions
Temporal design	2025 baseline + 2026 follow-up	2025 multiple dayparts; 2026 early evening	Introduce a temporal extension while retaining situated interpretation
Analytical logic	Within-phase thematic analysis + cross-phase comparison	2025 resident accounts read with 2026 photographic and field documentation	Identify recurrence, refinement, and divergence
Comparison logic	Interpretive rather than statistical or before-after	Different routes and participant profiles treated explicitly	Avoid causal overstatement while using the later walk to refine the sensory regime

**Table 2:** Cross-phase findings related to Cihangir's sensory urban transformation.

Dimension	2025 *sensewalking*	July 2026 couple *sensewalking* and cross-phase reading
Sensory organization	Strong contrasts between curated commercial nodes and quieter residential segments	The second route again shows concentrated commercial interfaces beside ordinary residential and infrastructural conditions; the regime remains nodal rather than continuous.
Commercial atmosphere	Cafe clusters, boutique spaces, pleasant smell associations, social visibility, and designed atmospheres	Outdoor seating, planter-defined edges, active frontages, and commercial street occupation remain prominent; curation persists as a visible spatial strategy.
Mobility and street pressure	Traffic, crowding, and high activity could shift vitality toward overload	Parked cars, taxis, scooters, bollards, narrow pavements, and pedestrian compression make the material basis of movement friction especially visible
Historical/material continuity	Historical traces and quieter streets supported familiarity and place attachment.	Historic masonry, religious structures, older facades, vegetation, and residential segments remain visible beside curated uses; continuity is overlain rather than erased.
Surface informality and heterogeneity	Perceptual discontinuities indicated that the neighborhood was not experienced as one coherent sensory field.	Graffiti, worn surfaces, ordinary service elements, and varied building conditions reinforce persistent material heterogeneity.
Belonging and exclusion	Some participants felt welcomed in certain nodes but expected to conform in others.	Curated interfaces coexist with constrained public movement and selective occupation of street edges; access is shaped both socially and spatially.
Sensory homogenization	Participants identified repetition and standardization in consumption-oriented areas.	The later photographic record shows that standardization is partial and spatially selective; homogenization is better understood as uneven intensification.
Sensory displacement	Familiar places could feel perceptually altered despite physical continuity.	Material continuity remains, but curated interfaces and mobility pressure reorganize the relative weight and meaning of familiar cues.
Participant lens	Resident-based group and solo accounts emphasized lived smell, sound, atmosphere, and belonging.	The paired architects foregrounded street section, material interfaces, historic traces, and spatial organization; the two forms of situated knowledge are complementary
Temporal interpretation	Provides the 2025 sensory baseline	The 2026 follow-up shows recurrence and refinement of the same mechanism, but not a controlled before-and-after change because route and participant profile differ.

Figure 2 summarizes the revised *sensewalking* procedure across both field phases, from participant preparation and sensory orientation to *in-situ* observation, documentation, dialogue, and post-walk reflection. The 2025 phase followed the original structured procedure of route orientation, sensory observation, photography, written notes, and reflection. The 2026 phase retained the same sensory focus while adding paired observation, a walking interview, video recording, and sound recording. It reflects methodological continuity between the two phases, along with the expanded multimodal and dialogic documentation introduced in 2026.

**Figure 2:**
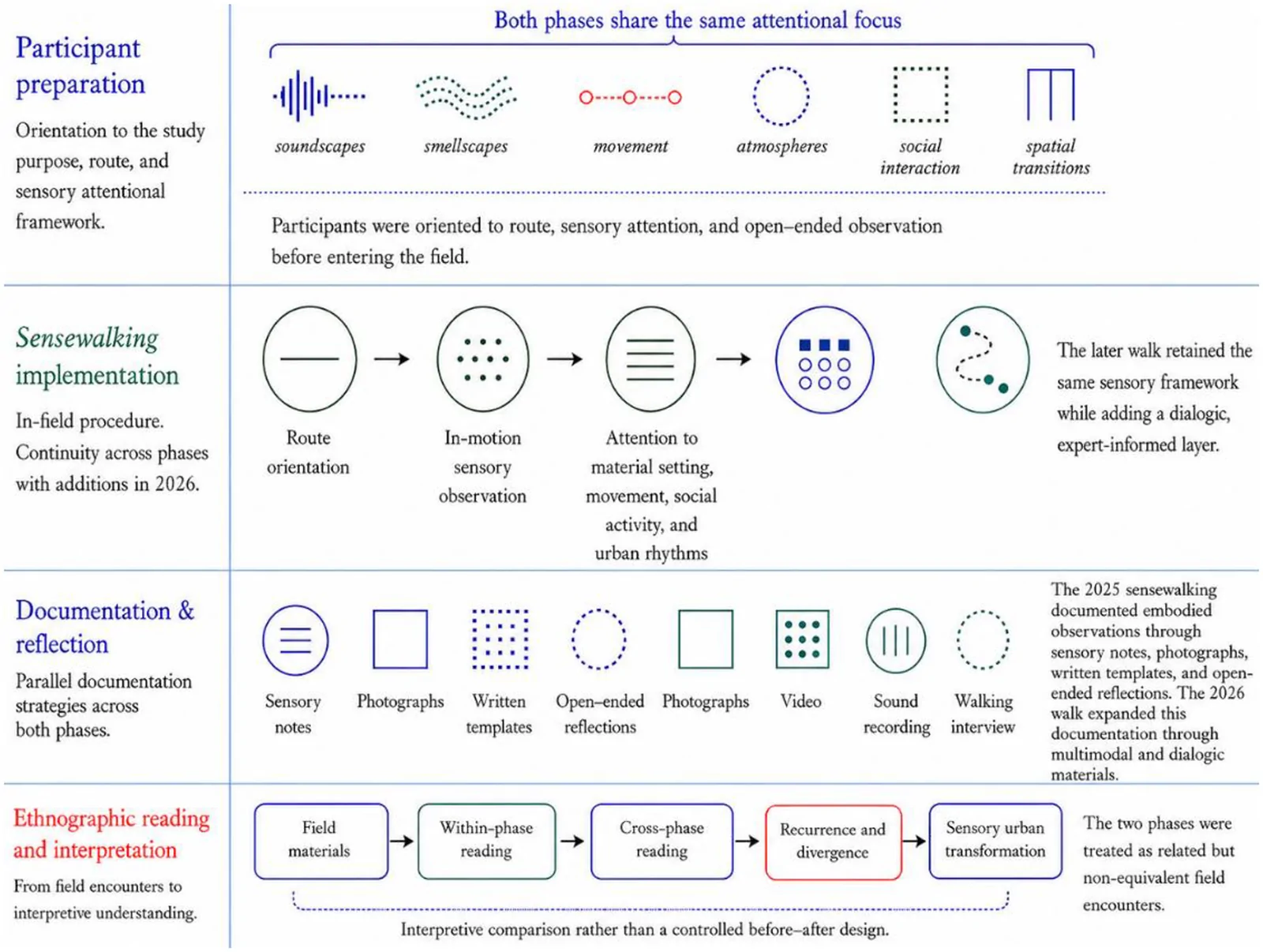
Multi-phase *sensewalking* procedure. Source: Own elaboration.

### 3.4 Analytical strategy: within-phase and cross-phase reading

After each field phase, the materials were organized by date, participant configuration, route, data type, and spatial encounter. The 2025 corpus consisted of written observations, completed templates, participant photographs, and route-based reflections. The 2026 corpus added a sequential set of 106 photographs together with video and sound recordings and a walking interview. The analytical structure distinguished the two phases before bringing them into relation, preventing the later expert walk from being merged uncritically with the earlier resident dataset.

We analyzed the 2025 data using thematic analysis through an iterative, inductive coding procedure informed by Braun and Clarke (2006). We reviewed field notes, participant reflections, observation templates, and visual materials repeatedly to become familiar with the dataset. We generated initial open codes from recurring references to sounds, smells, atmospheres, rhythms, social interactions, place attachment, spatial transitions, and perceptions of urban change. Related codes were grouped into broader analytical categories and refined into higher-order themes, including sensory plurality, sensory continuity, sensory commodification, sensory displacement, and the contrast between quieter residential segments and highly curated commercial nodes.

The 2026 follow-up was analyzed as a complementary field layer. The 106 photographs were reviewed as a sequence rather than as isolated illustrations and were coded for recurring material and spatial conditions that framed sensory experience. These included commercial curation and outdoor seating, planter-defined or managed street edges, mobility and parking pressure, pavement compression, historic and religious fabric, older residential facades, vegetation, graffiti and worn surfaces, and transitions between socially active and quieter segments. The walking-interview material was incorporated as *in-situ* interpretive excerpts rather than as a fully transcribed interview corpus. Statements were read thematically around topography, social heterogeneity, everyday street life, artistic memory, desirability, visual and media consumption, material continuity, and sensory overload. We translated the English excerpts from Turkish and lightly edited them for clarity while preserving their conversational register and analytical meaning. Where the source material was available as a thematic field-note formulation rather than a verbatim transcription, the findings identify it as a reconstructed participant reflection. We retained video and sound recordings as complementary multimodal materials. The cross-phase interpretation therefore draws directly on the photographic record, the walking-interview excerpts, and the documented conditions of the paired walk. We did not treat visible cafe settings or traffic conditions as direct proof of particular smells or sounds, but as spatial contexts that could be read alongside the 2025 olfactory and acoustic accounts.

Cross-phase analysis proceeded through an interpretive comparison of recurring and contrasting patterns rather than through statistical aggregation. The 2025 resident data provided detailed accounts of perceived smell, sound, atmosphere, belonging, and sensory discontinuity. The 2026 material provided a later, architecturally informed and strongly visual-material record of how curated commercial interfaces, infrastructure, historical traces, greenery, and everyday residential conditions coexisted within a second route. The comparison therefore asked whether the later encounter contradicted, confirmed, or refined the earlier themes. Particular attention focused on the spatial concentration of curation, the persistence of residual sensory anchors, the relationship between sociability and movement friction, and the distinction between neighborhood-wide homogenization and selective sensory intensification. Because the routes, participant profiles, and data formats differed, the comparison was treated as temporal and interpretive rather than as a controlled longitudinal measurement.

Figure 3 summarizes the revised analytical pathway from phase-specific raw materials to theoretical interpretation. It distinguishes the 2025 resident *sensewalking* corpus from the 2026 couple *sensewalking* corpus, shows within-phase coding, and indicates the cross-phase comparison through themes such as sensory curation, continuity, displacement, mobility pressure, historical traces, and selective intensification. The final conceptual step interprets these themes through the sensory urban transformation regime while retaining the methodological differences between the two phases.

**Figure 3:**
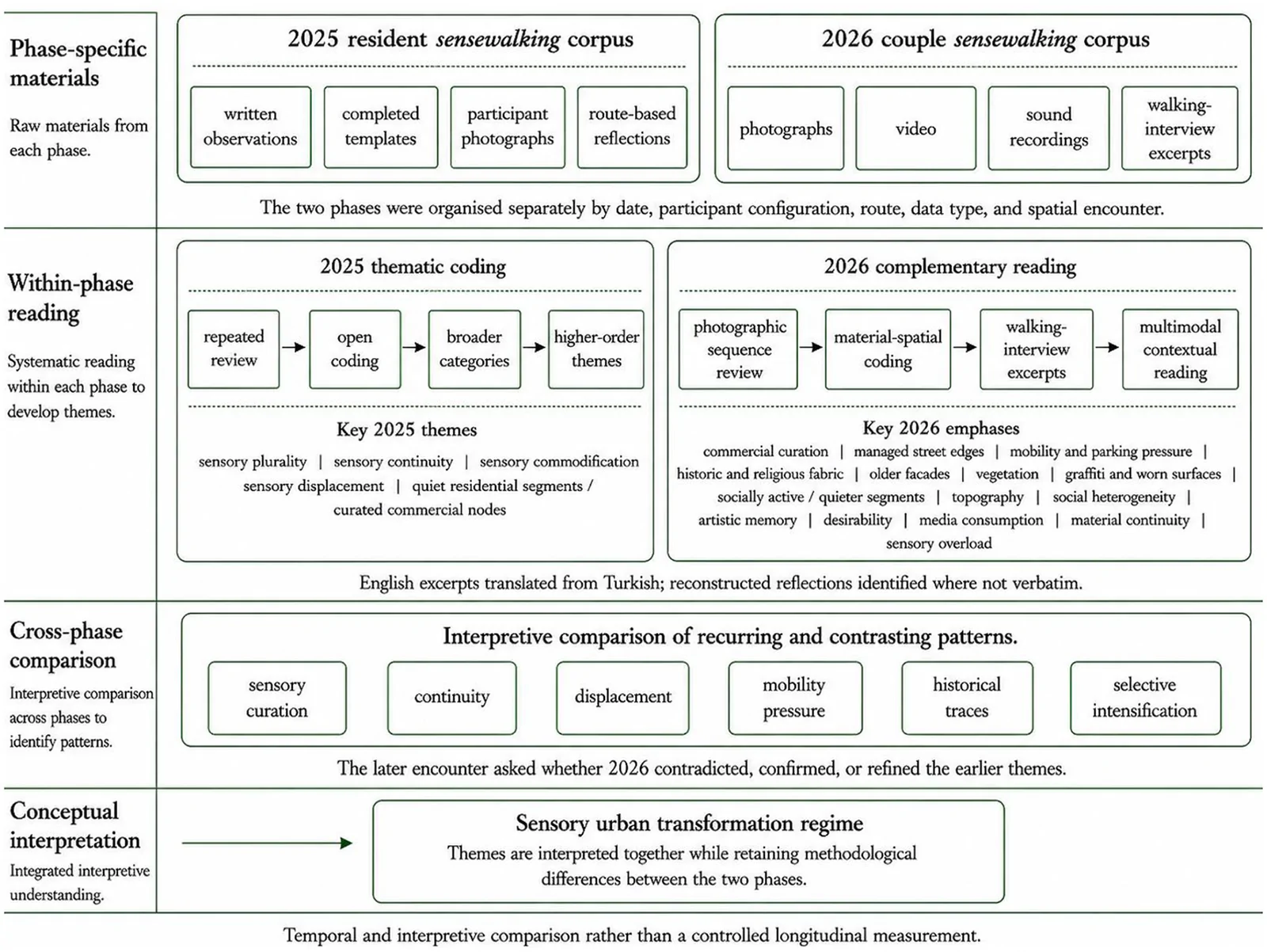
Analytical workflow for within-phase and cross-phase interpretation. Source: Own elaboration.

## 4 Findings

### 4.1 2025 *Sensewalking*: lived contrasts, curation, and sensory displacement

The 2025 *sensewalking* findings were interpreted within Cihangir's longer socio-spatial transformation. During the late Ottoman period, the district, shaped particularly by non-Muslim and Levantine populations, was characterized by architectural diversity and cultural richness (Uzun, 2003; Sandikçi and Ilkuçan, 2007; Yetiskul and Demirel, 2018; Uluengin, 2023). Political and demographic changes following the establishment of the Republic intensified migration, transformed property ownership, and deteriorated urban services, altering this socio-spatial profile from the 1950s onward (Eckardt and Wildner, 2015; Uluengin, 2023). The participants therefore walked through a neighborhood in which present-day sensory curation was already layered over older residential, architectural, and cultural traces.

Since the 1990s, Cihangir's revalorization has been strongly connected to its central location, housing stock, Bosphorus views, and proximity to Beyoglu's cultural life. These spatial advantages were reframed through sensory and symbolic imaginaries of the neighborhood as bohemian, artistic, and authentic (Uzun, 2003; Salgamcioglu and Ünlü, 2014; Demirel, 2015). Cafes, boutique spaces, and curated public environments introduced new *soundscape*s, visual aesthetics, smells, and social rhythms (see Figure 4).

**Figure 4:**
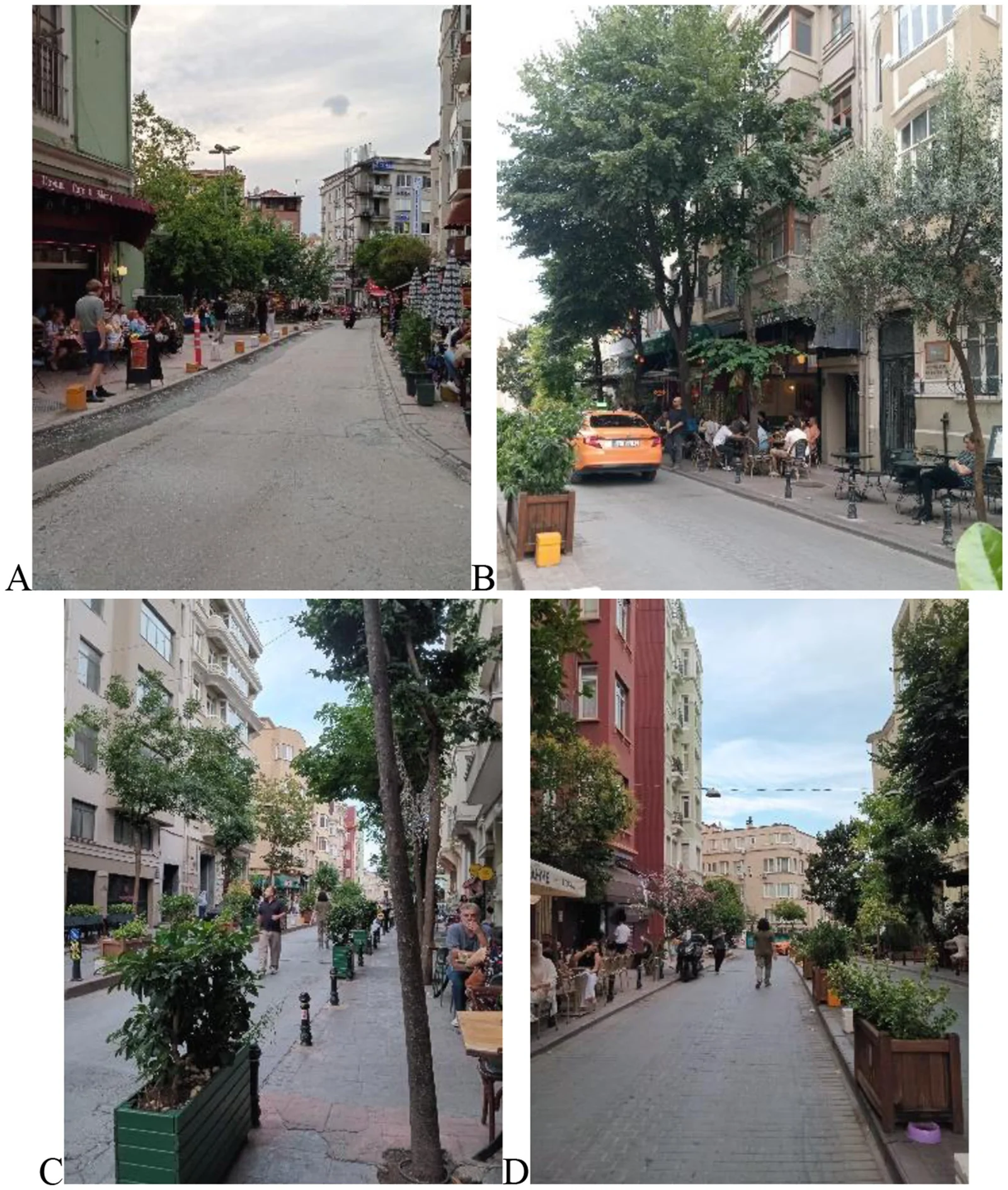
**(A–D)** Street-cafe settings and commercial interfaces in Cihangir. Source: Fieldwork materials.

From the mid-1990s onward, rising housing prices, rents, and changing user profiles also produced a pronounced class transformation (Demirel, 2015; Uzun, 2003, 2020). The 2025 *sensewalking* data made these transformations perceptible not only as economic change but as a reorganization of everyday atmosphere. In 2025, participants tended to evaluate places more positively when they perceived olfactory conditions as pleasant. High place evaluations frequently coincided with moderate-to-high smell evaluations, particularly in cafe and food-oriented areas. Higher sound evaluations were also often associated with positive place responses when acoustic activity was interpreted as social vitality rather than overload. Yet neither relationship was linear. Place experience remained multisensory and depended on the interaction of sound, smell, rhythm, crowd intensity, movement, and atmospheric transition.

The strongest ethnographic descriptions emerged when participants tried to explain why certain commercial environments felt attractive and, at the same time, strangely repetitive. One participant described the sensory work of curation in explicitly commercial terms: “*Many places feel carefully designed to create a particular atmosphere. Even the smells and sounds seem part of the experience being sold.”* This observation condensed an important contradiction. Market-driven transformation did not simply reduce sensory intensity; it reorganized which intensities were desirable, repeatable, and marketable, especially around cafe clusters and boutique-oriented nodes. The experience being sold was therefore not external to the neighborhood's sensory life but increasingly part of how that sensory life was organized.

The same contradiction appeared in the neighborhood's bohemian and artistic identity. Participants recognized Cihangir's cultural distinctiveness, but they also sensed that the image itself had become a form of lifestyle production. As one participant put it: “*The neighbourhood still has a bohemian image, but much of that identity now appears organised around consumption and lifestyle branding*.” The 2025 route made this spatial differentiation visible. Rainbow Steps emerged as a strong visual-atmospheric node; Alçakdam Yokuşu intensified bodily and kinesthetic experience through slope; Pürtelaş concentrated sound, smell, social activity, and commercial use; while Başkurt provided a quieter transition. Residential areas were generally described as slower and less acoustically intense, whereas cafe-dominated areas were associated with concentrated activity, visual presence, and social density. Cihangir's sensory regime therefore appeared more nodal than continuous.

Solo *sensewalker*s made the social consequences of these transitions especially tangible. Moving between quieter residential segments and more intensively curated settings, some participants described moments in which a street seemed to ask something of the person entering it. One participant expressed this ambivalence directly: “*There are places where I feel welcome and others where I feel like I am expected to conform to belong*.” Belonging was thus experienced not simply as access to space but as a bodily and perceptual negotiation with social codes, crowd density, atmosphere, and the expectations attached to particular places. Sensory pleasure and sensory pressure often occupied the same route.

The 2025 baseline ultimately showed that perceptual change could be experienced even where material continuity remained. A participant reduced this temporal disjunction to a simple but powerful sentence: “*The buildings may still be here, but the feeling of the neighbourhood has changed*.” This formulation became central to the study's understanding of sensory displacement. Familiar urban fabric could remain physically present while sounds, smells, rhythms, interactions, and the social meaning of everyday settings changed around it.

### 4.2 July 2026 couple *sensewalking*: memory, materiality, and everyday friction

The July 2026 couple *sensewalking* extended the study through a later field encounter rather than directly replicating the 2025 route. The two architects walked for 2 h between 18:00 and 20:00, applied the same sensory attention used in the earlier study, and documented the route through photography, video, sound recording, and a walking interview. The paired format created a dialogic field situation in which observation, memory, and professional interpretation unfolded while moving. The new phase is therefore treated as an expert-informed complement to, rather than a replacement for, the 2025 resident baseline.

Topography was among the first themes through which the later walk became more than a record of commercial change. The photographs repeatedly show streets that appear ordinary at close range but open suddenly toward distant water, sky, and the descending city. A reconstructed participant reflection captured the surprise of these openings: “*What surprises me in Cihangir is that the view does not announce itself. You turn an ordinary corner, sometimes in a street that seems completely unremarkable, and suddenly the city opens up in front of you. The Bosphorus appears almost accidentally*.” The same topography was felt through the body. Stepped streets did not operate only as picturesque images; they slowed movement, changed posture, and turned the sea view into something reached through effort. Another reconstructed reflection linked visual pleasure directly to kinesthetic experience: “*The stepped streets are one of the strongest experiences of Cihangir for me. You feel the slope through your body, but then the stairs suddenly frame the sea. The effort of moving downhill or uphill becomes part of the pleasure of seeing the city*.” These embodied and visual transitions are illustrated in Figure 5.

**Figure 5:**
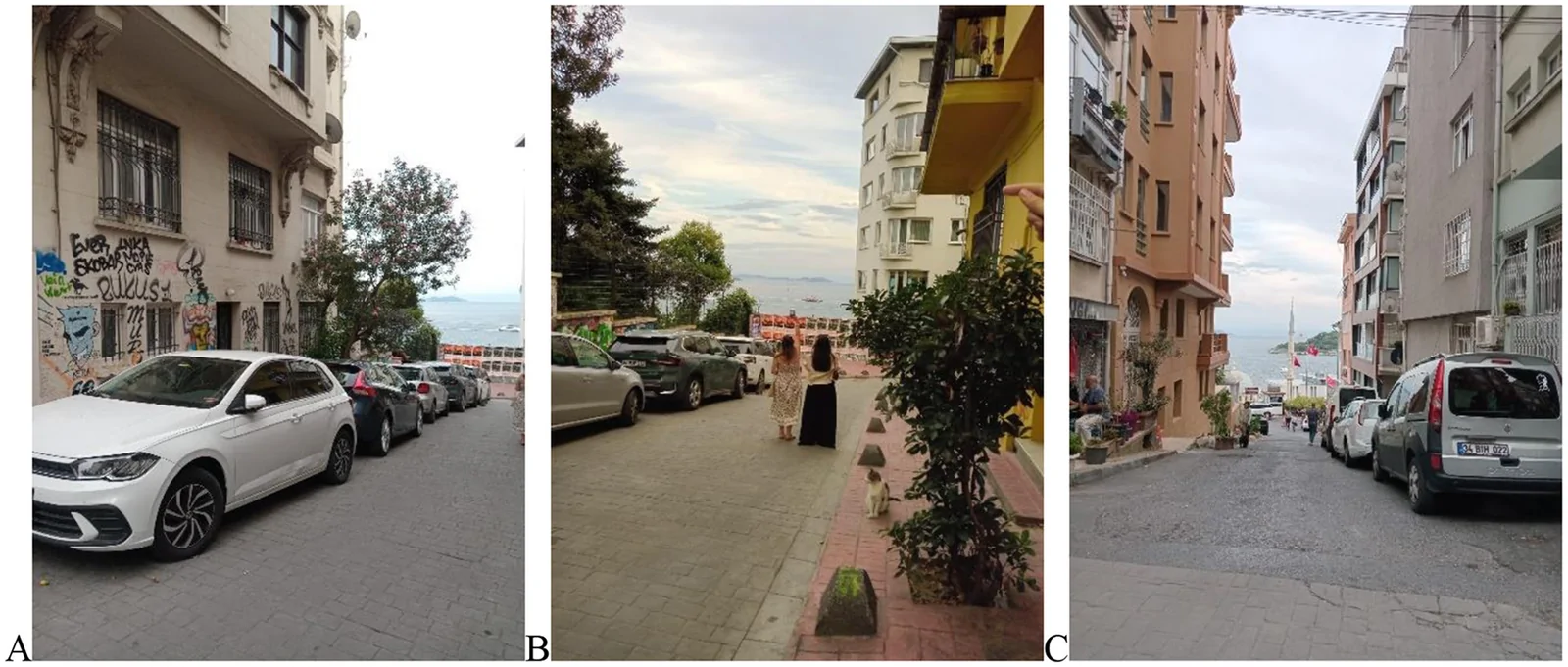
**(A–C)** Topography and visual openings encountered during the *sensewalking*. Source: Fieldwork materials.

Cihangir's attraction also lay in its unusual combination of centrality and separation. One translated and lightly edited reflection described this relationship in everyday terms: “*You can be on Istiklal Street in five minutes, but Cihangir still feels like a world of its own. That closeness to the centre without completely becoming part of it has always been one of the neighbourhood's strongest attractions*.”

The 2026 walk also resisted a simple reading of Cihangir as an enclave belonging to one class or cultural group. The paired reflections repeatedly returned to coexistence: long-term residents, creative workers, visitors, passers-by, service workers, and people drawn to the neighborhood's symbolic identity share the same narrow streets. A reconstructed reflection described this social mixture as part of the area's continuing vitality: “Cihangir seems to contain many different kinds of people at the same time. You can pass someone who has lived here for decades, a young creative worker, a tourist, and someone simply crossing the neighbourhood within a few minutes. That mixture is still part of what makes the area feel alive.”

A second reflection made the same point through the neighborhood's long association with cultural production while refusing to reduce Cihangir to the artistic elite: “*Cihangir has long attracted well-known actors, artists, and musicians, but the neighbourhood has never belonged to one social group alone. All kinds of people live here side by side. For me, that coexistence—the sense that very different lives can occupy the same streets—is still one of the things that makes Cihangir distinctive*.”

Everyday non-human life also entered the walking interview as part of this sense of inhabitation. Cats were not treated as a decorative emblem detached from the street, but as companions that alter the rhythm of walking and make public space feel unexpectedly domestic. A reconstructed reflection described the experience this way: “*The cats are almost part of the way you move through the neighbourhood. Once you step outside, one appears, then another, and sometimes they follow you along the street. They make the experience of walking here feel strangely domestic, as though the street itself is inhabited rather than passed through*.”

Memory became more complicated when the walkers spoke about Cihangir's artistic reputation. The neighborhood still carried prestige and recognizability, yet this prestige was often described as a trace of a social world that had changed. One walker reflected: “*You can still turn a corner in Cihangir and unexpectedly recognise a well-known face. But I remember it as a place where the artistic community felt more deeply rooted. The neighbourhood still carries a sense of refinement, although perhaps some of that now survives more in memory than in everyday life*.”

Another participant located that change more explicitly within gentrification and rising property values: “*Cihangir was once a place where people from the arts could actually afford to live. From the mid-2000s onward, rising property values turned the neighbourhood into a social-status symbol. The arts crowd is still visible today, but many no longer live here. What once developed organically as an artistic habitat has gradually become something closer to a simulation of one*.” This sense of loss was not total. The photographs document older residential facades, masonry walls, religious structures, mature vegetation, worn surfaces, and shopfronts that continue to interrupt the curated commercial image. One walker rejected the more familiar shorthand of cats and cafes and located the neighborhood's continuity elsewhere: “*People talk about the cats and the cafes, but for me the most beautiful part of Cihangir is still those shops that have kept their old design. You pass them and, for a moment, the neighbourhood feels connected to an older version of itself*.” Figure 6 shows how historical and everyday material traces continue to function as perceptual anchors within the transformed neighborhood.

**Figure 6:**
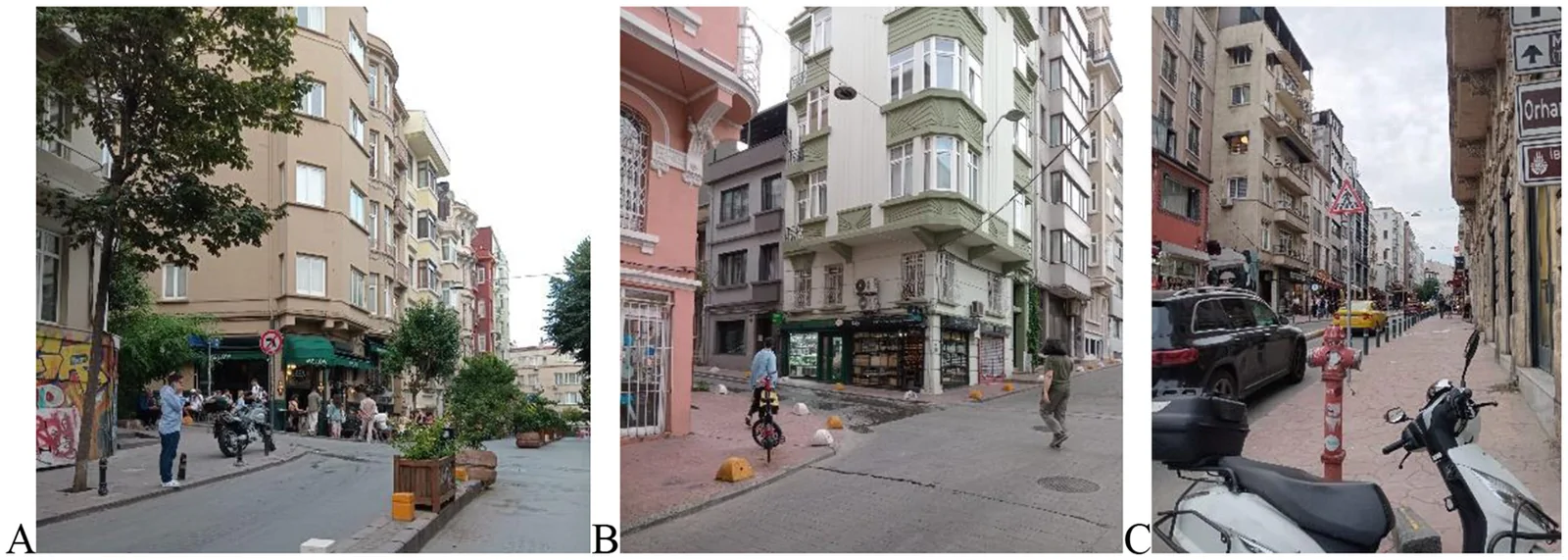
**(A–C)** Residual material continuity in contemporary Cihangir, including older facades, shopfronts, and street edges. Source: Fieldwork materials.

These material anchors coexist with a powerful market of desire. The walking interview repeatedly returned to the idea that Cihangir's name itself has acquired exchange value, so that the wish to inhabit the neighborhood can become detached from the everyday quality of inhabiting it. One participant stated: “*Everyone wants a piece of Cihangir now. The desire to live here has become a market in itself. At some point, the idea of Cihangir began to carry almost as much value as the neighbourhood itself*.” Media production was described as one mechanism through which this symbolic value is continuously renewed. The neighborhood's streets function not only as settings of everyday life but also as recurring visual material for television and cinema: “*It feels as though almost every week you run into a television or film shoot somewhere in Cihangir. After a while, you realise that the neighbourhood is not only lived in; it is constantly being used as an image of itself*.” The same visibility was experienced at the scale of everyday photography. Certain stairs, facades, views, and corners appeared to operate as informal stages for continuous image-making. A reconstructed participant reflection described this visual pressure as extending across the day: “*Some places no longer feel quiet even in the morning. By the time you arrive, people are already photographing themselves, the stairs, the facades, the view. In the evening the same thing happens again. It sometimes feels as if certain corners of the neighbourhood have become stages that are continuously being photographed*.”

The more critical reflections placed these contemporary forms of desirability against a longer personal memory of the neighborhood. One participant described the early 2000s as a period when Cihangir's bourgeois-bohemian identity remained less comfortable, less polished, and therefore more distinctive: “*In the early 2000s, Cihangir was genuinely bourgeois-bohemian, without much comfort—which was part of its character. As it became more popular toward the 2010s, it flourished but also experienced harsh gentrification. Something distinctive still survived for a while. Today, however, it reminds me of someone who had a beautiful youth but did not age well*.”

The photographs and the walking interview also made the material costs of contemporary intensity difficult to separate from its vitality. Outdoor seating, cafe and retail frontages, planters, parked cars, taxis, scooters, bollards, waste containers, narrow pavements, and constrained pedestrian passages repeatedly occupy the same limited street section. One participant's account brought smell, noise, dust, rents, and movement pressure into a single lived scene: “*The neighbourhood now feels overrated and increasingly compressed. The streets are narrow and crowded, rubbish containers overflow, and in places the air smells of garbage and urine. You sit in a cafe watching rubbish trucks, construction vehicles, and street vendors pass by, surrounded by noise, dust, and traffic. I genuinely do not understand who pays these rents anymore, or what they are paying them for*.” The spatial conditions underlying this account—curated sociability, service infrastructure, and mobility pressure—are visible in Figure 7.

**Figure 7:**
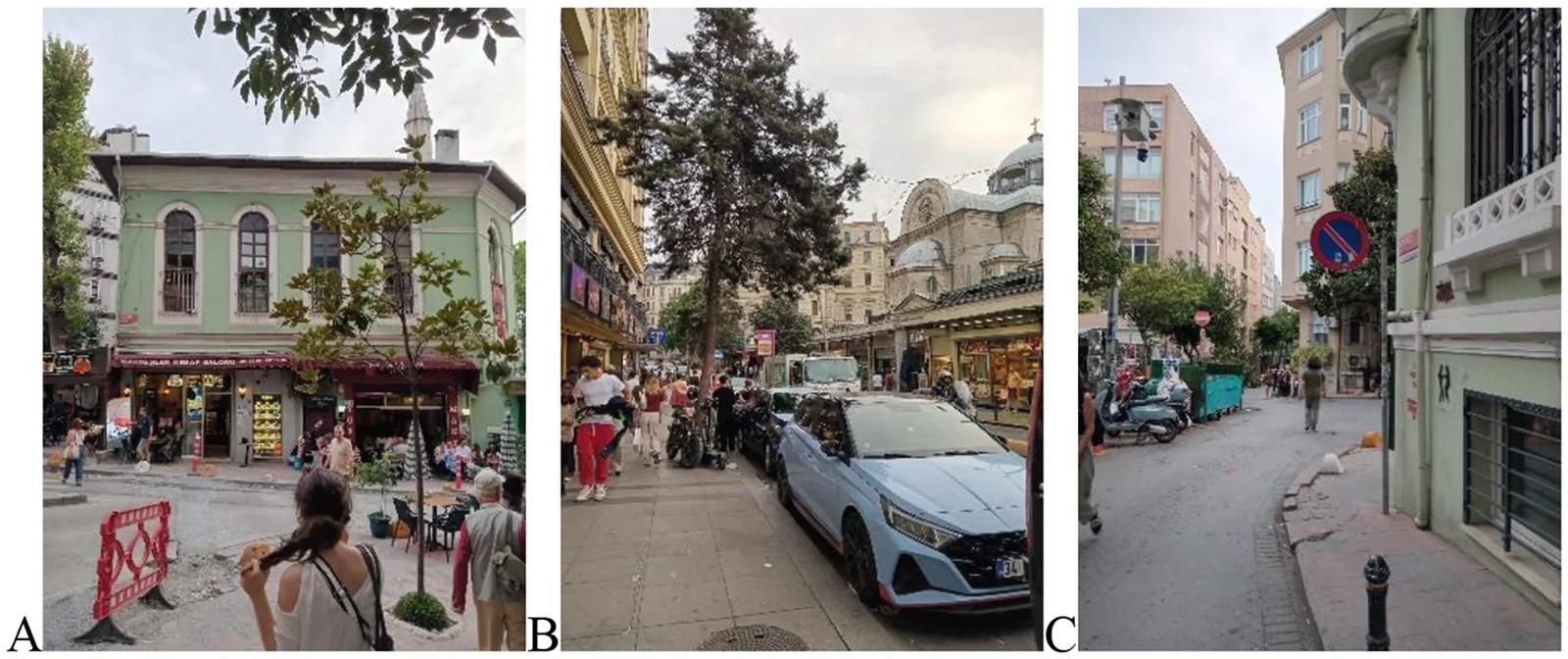
**(A–C)** Service infrastructure, mobility pressure, and compressed street sections encountered during the *sensewalking*. Source: Fieldwork materials.

The 2026 material therefore complicates any interpretation of fully homogeneous sensory experience. Commercial curation remains visible and powerful, but it is layered with topographic surprise, social heterogeneity, everyday animal life, historical and residential traces, old shopfronts, media visibility, waste, traffic, and infrastructural friction. The later walk suggests that Cihangir's transformation is felt less as the replacement of one coherent atmosphere by another than as the intensified coexistence of competing atmospheres within the same compressed urban field.

### 4.3 Cross-phase synthesis: continuity, reweighting, and selective intensification

Reading the two field phases together demonstrates continuity of mechanism rather than a simple linear intensification of gentrification between 2025 and 2026 (see Tables 3 and 4). In both phases, market-oriented sensory production was concentrated at particular nodes and interfaces and interrupted by quieter, residential, historical, infrastructural, or topographic conditions. The 2025 resident accounts described these differences through smell, sound, atmosphere, belonging, and bodily transition; the 2026 walk made comparable differentiation visible through street-edge occupation, material continuity, visual openings, media use, and the friction of everyday mobility.

**Table 3:** Revised analytical characteristics of the sensory regime in Cihangir.

Dimension	Revised Cihangir sensory regime	Cross-phase evidence
Urban transformation path	Predominantly market-driven and fragmented transformation with selective sensory intensification	2025 consumption clusters and 2026 concentrated commercial street edges
Dominant governance configuration	Market actors supported by local initiatives, small businesses, and symbolic place-making	Commercial curation appears through multiple localized interfaces rather than one centralized design
Sensory character	Curated at key nodes, materially layered, and unevenly homogenized	2025 nodal intensity + 2026 coexistence of commercial curation, infrastructure, historic traces, graffiti, and greenery
*Soundscape*	Social vitality and traffic pressure coexist; sound is partly commodified in consumption-oriented nodes	2025 participant sound evaluations; 2026 sound recording collected, while photographic evidence documents activity and traffic contexts
*Smellscape*	Food- and coffee-oriented olfactory value is concentrated around consumption settings	2025 smell/place associations; 2026 photographs document cafe/food interfaces but are not treated as direct olfactory evidence
Atmosphere	Lifestyle-oriented and curated in selected nodes, but interrupted by residential, infrastructural, and historical conditions	Both phases show abrupt or visible transitions between active and quieter segments.
Cultural production	Authenticity branding coexists with residual material informality and historical identity.	Bohemian representation in 2025; graffiti, older fabric, religious structures, and everyday street conditions remain visible in 2026
Experience of belonging	Produced through social activity, lifestyle affiliation, and residual familiarity	2025 place attachment and sociability; 2026 persistence of residential and historical anchors
Experience of exclusion	Produced through selective atmosphere, social codes, and movement friction	2025 conformity/exclusivity accounts; 2026 narrow pavements and shared street-edge pressure
Main form of sensory displacement	Reweighting of familiar sensory cues rather than complete erasure	Familiar material traces persist while curated interfaces and new rhythms alter how the neighborhood is experienced
Overall sensory regime	Market-oriented, commodified, selectively intensified, and materially layered	Cross-phase recurrence of nodal curation combined with persistent heterogeneity

**Table 4:** Revised gentrification and sensory transformation framework in Cihangir.

Transformation logic and core urban mechanisms	Governance structure	Main actors	Social outcomes	Spatial outcomes	Basic sensory outcomes
Market-driven transformation through branding and consumption	Market-dominant fragmented governance; consumption-led micro-governance	Investors, small businesses, consumers, local initiatives	Accelerated class transition; selective belonging; experiential alienation	Consumption clustering; spatial concentration	Curated *soundscape* and *smellscape*; atmosphere commodification; repeated sensory codes
Historically sedimented transformation through memory retention	Embedded cultural continuity within fragmented governance	Long-term residents, local organizations, everyday users	Place attachment; cultural memory; residual familiarity	Historic and residential traces remain within transformed fabric	Historical cues, greenery, familiar routes, food-smell memory, sensory continuity
Experience-oriented transformation through lifestyle practices	Consumption-centered governance	Cafes, boutiques, patrons, urban users	Lifestyle-based inclusion/exclusion; attraction and fatigue	Curated public interfaces; social concentration	Designed atmospheres; concentrated sensory pleasure; altered rhythms and sociability
Selective sensory intensification through street-edge occupation and mobility pressure	Distributed street-level micro-governance	Businesses, pedestrians, residents, drivers, service actors	Sociability combined with access friction and contested public use	Narrow pavement compression; parking and seating overlap; uneven street interfaces	Partial homogenization; sensory overload; material heterogeneity; residual continuity

The participant voices sharpen this continuity. In 2025, a resident described places as “carefully designed” so that even smells and sounds seemed to belong to an experience being sold. In 2026, one architect described an older artistic habitat as having become “something closer to a simulation of one.” The two formulations emerge from different field moments and participant positions, yet both identify a shift from lived distinctiveness toward the organized reproduction of distinctiveness as an urban value.

The later fieldwork makes it harder to describe this process as neighborhood-wide homogenization. The 2026 photographs and walking interview retain a dense field of counter-signals: old shopfronts, worn facades, religious structures, mature vegetation, cats, stepped streets, unexpected sea views, mixed social presence, and ordinary service infrastructure. Graffiti formed part of the neighborhood's visible and tactile material field (see Figure 8). These elements do not sit outside gentrification; they coexist with it, sometimes becoming part of the very authenticity through which Cihangir is valued.

**Figure 8:**
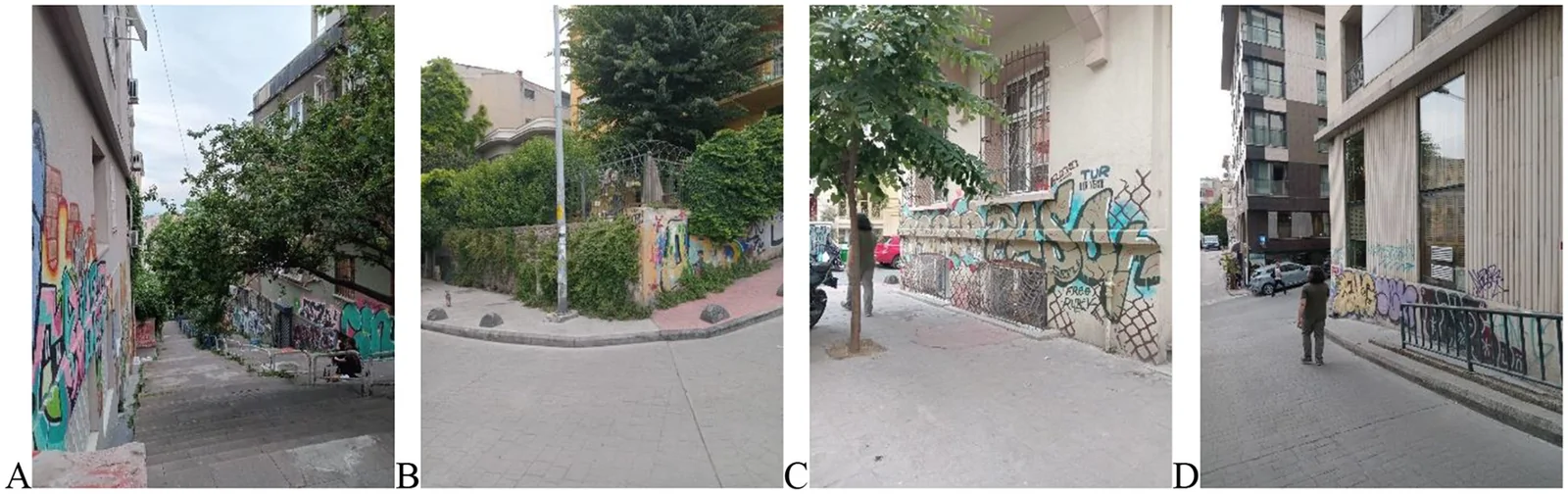
**(A–D)** Graffiti on older and newer building facades in Cihangir. Source: Fieldwork materials.

This refinement also changes how we understand sensory displacement. The 2025 statement that “the buildings may still be here, but the feeling of the neighbourhood has changed” describes displacement through material continuity and perceptual change. The 2026 metaphor of a place that “had a beautiful youth but did not age well” adds a more explicitly temporal and affective register. Together they suggest that displacement can occur through a reweighting of the perceptual field: familiar cues remain, but their relative prominence, meaning, and social ownership change.

Belonging is similarly double-sided. The 2025 participant who felt welcomed in some places but expected to conform in others described selective belonging from within the resident-based walks. The 2026 material, by contrast, emphasized both social heterogeneity and a powerful desire to possess the Cihangir identity. Coexistence and exclusion are therefore not opposites. They can be produced simultaneously when different people share the same streets while certain commercial, aesthetic, and economic codes become increasingly dominant.

The two phases also produce different but complementary forms of situated knowledge. Residents in 2025 foregrounded affect, smell, sound, familiarity, discomfort, and attachment. The architects in 2026 foregrounded street section, topography, old shopfronts, material interfaces, artistic memory, symbolic value, media visibility, and the spatial conditions of overload. The difference is analytically productive: it shows that a sensory regime is encountered not only through place but also through biography, profession, memory, walking configuration, and the medium through which experience is recorded.

Cihangir is consequently interpreted as a market-oriented but materially and socially layered sensory regime, characterized by selective curation, partial homogenization, residual continuity, symbolic desirability, mobility pressure, and sensory displacement. The later phase does not prove a controlled before-and-after change, because route and participant configuration differ; instead, it refines the spatial and human meaning of the mechanism identified in 2025.

## 5 Discussion

### 5.1 Beyond the visual: gentrification as lived sensory reorganization

The two phases of *sensewalking* demonstrate that gentrification cannot be understood only through economic revaluation, demographic change, or spatial restructuring. Cihangir's transformation was encountered through changing sounds, smells, rhythms, atmospheres, movement conditions, social practices, views, street edges, and material interfaces. The ethnographic significance of these changes lies precisely in their ordinariness: they become noticeable while climbing a stair, turning a corner, sitting at a cafe, passing an old shop, negotiating a rubbish container, or recognizing that a familiar street now asks the body to move differently.

The 2025 and 2026 field encounters therefore extend displacement beyond physical relocation. Sensory displacement concerns changes in the perceptual conditions through which familiarity, attachment, and belonging are produced. The persistence of buildings, old shopfronts, vegetation, religious structures, or even familiar routes does not guarantee continuity of experience. What matters is how these elements are weighted within a changing field of commercial sound, smell, visual production, crowd density, rents, movement pressure, and social expectation.

Topography is important here because it resists an exclusively commercial reading of Cihangir. The 2026 reflections on stepped streets and sudden Bosphorus views show how the neighborhood still offers sensory experiences that are not reducible to consumption. The body labors on the slope; the view appears unexpectedly; an ordinary corner can open into a wider urban horizon. These moments help explain why strong attachment can persist even in a neighborhood whose symbolic and economic value has become increasingly commodified.

Social heterogeneity produces a similar tension. The later walkers described Cihangir as a place in which very different lives continue to occupy the same streets, while the 2025 residents described some curated environments as socially selective. The coexistence of these accounts suggests that diversity and exclusion operate at different scales. A neighborhood may remain socially mixed in its everyday movement while particular cafes, streets, and aesthetic codes organize more selective forms of comfort and belonging.

The material compression visible in 2026 helps explain why vitality and fatigue repeatedly appeared together. Outdoor seating, pedestrians, parked vehicles, taxis, scooters, waste containers, service activity, and commercial display compete within a narrow public realm. The participant who described drinking coffee amid rubbish trucks, construction vehicles, noise, dust, and traffic did not separate consumption from infrastructure; they experienced both in the same sensory scene. Curation and overload are therefore interdependent dimensions of the same experience-oriented urban regime.

The same holds for cultural production. Cihangir's long association with artists, actors, and musicians continues to generate symbolic capital, but the interview material suggests that the relationship between artistic presence and artistic residence has changed. The phrase “simulation of an artistic habitat” captures a central contradiction: the image of bohemian life can remain highly visible even as the economic conditions that once allowed it to reproduce itself locally become harder.

Film and television shoots, along with the continuous photographing of stairs, views, and facades, extend this process into visual circulation. Cihangir is not only represented from outside; it is repeatedly used as an image of itself. This media visibility strengthens desirability and recognition, but it can also reorganize everyday space around the expectations of visitors, audiences, and lifestyle consumption. The visual is therefore not separate from the sensory regime; it interacts with sound, movement, crowding, and the performance of social identity.

Yet the old shops, worn surfaces, animals, historic fabric, and quieter residential segments prevent this image from becoming total. The participant who valued the old shop designs more than “the cats and the cafes” points to a form of everyday heritage that is neither monumental nor fully curated. Such interfaces operate as sensory anchors: they connect present movement to remembered urban forms and allow continuity to be felt at the scale of a doorway, sign, facade, or habitual route.

This layered condition requires a more precise use of sensory homogenization. The 2025 accounts of repetition remain important because they capture how curated environments can feel standardized at the level of lived experience. The 2026 material does not invalidate those accounts; it shows that standardization is spatially partial. Homogenization is better understood as an uneven process of sensory coding and selective intensification, in which certain commercial and symbolic cues become more prominent without erasing the heterogeneous material field around them.

The humanistic dimension of the findings lies in this simultaneous presence of attraction and loss. Cihangir can still feel like “a world of its own” only minutes from Istiklal Street, while also being remembered as a place whose earlier artistic life has become more distant. The neighborhood is neither simply authentic nor simply commodified, neither socially open nor entirely exclusive. People live through these contradictions, and gentrification becomes perceptible precisely where they are felt in the body and narrated through memory.

### 5.2 Between memory and curation: cross-phase interpretation

The most important result of reading 2025 and 2026 together is continuity of mechanism rather than evidence of a simple linear intensification over time. In both phases, curated and consumption-oriented environments clustered at particular interfaces and were interrupted by quieter, residential, historical, infrastructural, or topographic conditions. The later walk therefore confirms the persistence of a nodal sensory regime, but the different route and participant profile prevent reading the phases as a controlled before-and-after measurement.

What changes across the phases is the language through which transformation becomes intelligible. The 2025 participants spoke from lived familiarity and described atmosphere through attraction, discomfort, smell, sound, and belonging. The 2026 architects added longer temporal memories and a professional attentiveness to street sections, facades, topography, and material continuity. Their metaphor of Cihangir as someone who “had a beautiful youth but did not age well” does not function as an objective history of the neighborhood; it is valuable because it reveals how urban change is moralized, remembered, and emotionally condensed in everyday speech.

This distinction also refines sensory displacement. Sensory displacement is not necessarily a movement from familiarity to total unfamiliarity. Older buildings, old shops, religious structures, vegetation, cats, residential rhythms, and sea views can remain present while curated commercial practices, rising symbolic value, media visibility, and movement pressures alter the relative dominance of sensory cues. Sensory displacement can therefore be understood as a reweighting of the perceptual field: some cues become commercially amplified, some are backgrounded, and others survive as anchors of memory and attachment.

The idea that “everyone wants a piece of Cihangir” makes this reweighting inseparable from symbolic capital. Desire for the neighborhood is not merely a response to housing or location; it is also desire for an urban identity. Proximity to Istiklal, association with artists and celebrities, recurring film production, picturesque stairways, sea views, cafes, and an inherited bohemian reputation combine to produce a value that exceeds the material dwelling itself. Gentrification is therefore reproduced through the circulation of an image as much as through the circulation of property.

At street level, however, this symbolic value is mediated through small and often mundane spatial decisions. Businesses, residents, pedestrians, motorists, service actors, planters, seating, parked vehicles, and waste infrastructure collectively shape the limited public realm. The 2026 images make this micro-governance visible, while the interview material gives it affective force through descriptions of compression, odor, noise, dust, crowding, and difficulty of movement. Sensory stratification operates through both symbolic codes and material access: who feels appropriate in a space, which sensory cues dominate, and how easily different users can move, pause, or remain.

The comparison therefore supports a relational rather than deterministic account of neoliberal sensory production. Market-oriented curation is powerful, but it is never the only force present. Residual material continuity, social heterogeneity, everyday animal life, old commercial interfaces, topography, and memory continue to complicate the neighborhood's sensory field. These elements can resist standardization, be incorporated into authenticity branding, or do both.

Methodologically, the multi-phase design shows the value of keeping different forms of situated knowledge in tension. The resident group and solo *sensewalking*, and the architect-led couple *sensewalking*, should not be collapsed into a single homogeneous dataset. Their difference is the point: the first phase reveals how Cihangir is lived through familiarity and everyday perception, while the second shows how the same urban field can be re-read through dialogue, professional knowledge, visual documentation, and retrospective memory. Cross-phase interpretation thus makes the sensory regime more precise without overstating temporal causality.

Cihangir's transformation has reflected a rapid, market-oriented restructuring in which aestheticization and lifestyle consumption play a central role (Yetiskul and Demirel, 2018). The two field phases extend this account by demonstrating that restructuring also operates through sensory production and through the spatial organization of everyday interfaces. Cafe-based sounds and smells, commercial display, outdoor seating, crowd rhythms, and lifestyle-oriented sociability are central to this process, but the 2026 material shows that these conditions remain interwoven with parking, traffic, historic fabric, greenery, graffiti, and residential use. The neoliberal sensory regime is therefore produced through selective concentration rather than complete material replacement.

The analysis demonstrates that gentrification cannot be understood as a uniform, linear, or purely material process. Under neoliberal urban conditions, Cihangir's historical and everyday sensory qualities are selectively reorganized into commodified urban experiences. The 2025 and 2026 data together suggest that the most appropriate description is not a total sensory standardization but an uneven reordering in which curated nodes gain perceptual and social prominence. This perspective calls for urban policy frameworks that consider sensory diversity, everyday accessibility, and the coexistence of commercial vitality with non-commercial forms of urban life.

Sensory transformation was also embedded in changing class relations and urban social boundaries. In 2025, participants connected curated atmospheres to selective belonging and moments of alienation. In 2026, the photographic record showed how these atmospheres were materially supported by the occupation and management of narrow street edges, where commercial seating, pedestrians, vehicles, and street furniture competed for limited space. Sensory stratification therefore operates through both symbolic codes and material access. Urban inequality can be reproduced through who feels socially appropriate in a space, which sensory cues dominate, and how easily different users can move, pause, or remain within the public realm.

### 5.3 Methodological limitations and future research

Despite this study's methodological approach, which conceptualizes gentrification as a sensory urban transformation regime, several limitations warrant acknowledgment. First, *sensewalking* remains inherently selective in capturing the full complexity of multisensory urban experience. Sensory perception is situational and filtered through individual cognitive, emotional, professional, biographical, and socially situated conditions (Barwick-Gross et al., 2025). Urban sensory environments are also unstable, shaped by weather, crowd density, commercial activity, traffic, and momentary rhythms. The 2025 and 2026 findings should therefore be interpreted as situated field encounters rather than fixed representations of Cihangir's sensory regime.

The participant structure also requires caution. The 2025 phase involved 20 local adult residents aged 25–69, whereas the 2026 follow-up involved two architects living in Istanbul who were academically familiar with Cihangir's transformation. Taken together, the study engaged 22 participants, but these participants do not constitute a homogeneous sample. The architects add an expert-informed, dialogic perspective rather than demographic breadth, and their professional training may direct attention to spatial and material conditions that other users would interpret differently. The phases are therefore analytically complementary but not directly equivalent.

The 2026 follow-up partially addresses the original fieldwork's temporal limitation by extending the case 1 year beyond the 2025 walks, but it does not constitute a longitudinal or before-and-after design. The second phase used a different route, only two participants, and a single early-evening time window (18:00 to 20:00). The 106 photographs provide a substantial visual-material record, but photographs cannot directly capture olfactory or acoustic experience. The walking-interview material was used thematically through selected translated and lightly edited excerpts rather than through full transcript-level coding, while video and sound recordings remain only partially analyzed. A fuller future analysis could examine the interview, video, and sound materials systematically through transcription, acoustic categorization, temporal sequencing, and direct cross-referencing with specific route segments. Seasonal variation and longer-term change also remain outside the present design.

The other limitation concerns spatial scope. The study focuses on one highly specific inner-city neighborhood with a distinctive historical, political, and socio-cultural trajectory. Cihangir provides a productive framework for analyzing fragmented and market-driven transformation, but its sensory regime cannot be directly generalized to all gentrification contexts. Future studies could repeat comparable paired or group walks along matched routes, include displaced residents, temporary users, commuters, and other professional groups, and combine photographic, acoustic, olfactory, and interview materials within a more explicitly longitudinal framework.

## 6 Conclusion

This study, through two phases of sensory-ethnographic fieldwork in Istanbul's Cihangir neighborhood, demonstrated that gentrification cannot be adequately explained as a process confined to economic revaluation or spatial restructuring. Rather, it emerged as a multi-dimensional urban transformation regime shaped through interactions among market forces, localized governance, civil initiatives, cultural production, everyday practices, memory, and the sensory organization of street-level life. The 2025 resident *sensewalking* and the July 2026 couple *sensewalking* together revealed that gentrification operates not only through the transformation of built environments and social structures but also through the selective reconfiguration of sensory urban experience.

Returning to the two research questions, the study showed, first, that market-driven gentrification transforms Cihangir beyond visual dimensions by reorganizing *soundscape*s, *smellscape*s, rhythms, atmospheres, movement conditions, social interaction, symbolic visibility, and the spatial interfaces through which everyday life is experienced. Second, the cross-phase reading revealed both continuity and refinement. The 2025 participants identified cafe-oriented curation, olfactory and acoustic place value, nodal sensory intensity, and selective belonging. The 2026 photographic and walking-interview material again documented concentrated commercial and social interfaces, but also made topographic pleasure, social heterogeneity, artistic memory, old shopfronts, media visibility, historic and religious traces, greenery, everyday animal life, waste, traffic, and ordinary infrastructure especially visible.

The comparison therefore does not support a simple narrative in which Cihangir became uniformly more homogenized between 2025 and 2026. Because the routes and participants differed, the phases cannot establish linear temporal change. Instead, the later encounter confirms the durability of the mechanism identified in 2025 while refining its spatial form. Cihangir's sensory regime is best understood as market-oriented, selectively intensified, and materially layered. Curated atmospheres gain prominence at particular nodes and interfaces, while heterogeneous material and sensory anchors persist around them.

The case also reframes displacement. Rather than focusing solely on physical relocation, the findings emphasize sensory displacement as a condition in which the relative weight and meaning of familiar sensory cues change. Residents may remain physically present, and historical or everyday traces may remain materially visible, while commercially amplified sounds, smells, rhythms, social codes, and movement pressures alter how the neighborhood feels. Sensory displacement is therefore not necessarily the erasure of familiarity but its reorganization within a changed perceptual hierarchy.

From a theoretical perspective, the study contributes to urban sociology by conceptualizing gentrification as a sensory urban transformation regime and by refining sensory homogenization as partial and uneven. The Cihangir case shows that neoliberal sensory production can operate through selective intensification: particular commercial edges, social nodes, and lifestyle-oriented settings are curated and repeated while other residential, historical, infrastructural, and informal conditions remain perceptible. This coexistence explains why pleasure, authenticity, overload, attachment, exclusivity, and alienation can be experienced simultaneously within the same neighborhood.

Methodologically, the multi-phase design shows the value of reading different walking configurations and recording media together without treating them as equivalent. Group and solo *sensewalking* generated resident-based accounts of lived sensory experience, while the later couple *sensewalking* added a dialogic and architecturally informed perspective together with extensive photographic, video, and sound documentation. Photographs proved especially useful for identifying the material organization of sensory conditions, provided that they were not treated as direct substitutes for smell, sound, or embodied perception. Future analysis of the walking interview, video, and sound recordings can further deepen this multimodal approach.

From a policy perspective, the findings underscore the limitations of approaches that prioritize spatial improvement while neglecting experiential and sensory dimensions of urban life. Urban policy and planning should consider not only physical renewal, land-use change, or visual quality, but also the distribution of sensory intensity, the accessibility of street edges, the coexistence of commercial and non-commercial uses, and the preservation of familiar material and sensory anchors. The single-case, non-equivalent two-phase design limits generalization, but it also points toward more explicit longitudinal and multimodal research on sensory change.

This article demonstrates that gentrification is not merely the transformation of physical space, but a process through which social relations, power structures, sensory cues, and everyday urban experiences are continuously reordered. In Cihangir, this reordering occurs through the concentration of curated atmospheres within a materially heterogeneous neighborhood. Urban environments should therefore be understood as sensory and experiential ecosystems in which commodification and continuity, attraction and pressure, familiarity and displacement coexist. Foregrounding these relations provides a more precise understanding of how neoliberal urban transformation is experienced, perceived, and negotiated in everyday life.

## Data Availability

The raw data supporting the conclusions of this article will be made available by the authors, without undue reservation.
